# Stability of Purple Corn Anthocyanin Encapsulated by Maltodextrin, and Its Combinations with Gum Arabic and Whey Protein Isolate

**DOI:** 10.3390/foods12122393

**Published:** 2023-06-16

**Authors:** Wei Deng, Xiaoyi Li, Guoqiu Ren, Qingmei Bu, Yanye Ruan, Ying Feng, Bin Li

**Affiliations:** 1 Food College, Shenyang Agricultural University, Shenyang 110866, China; dw1998@stu.syau.edu.cn (W.D.); lxysynydx@stu.syau.edu.cn (X.L.); rgqsy@stu.syau.edu.cn (G.R.); b15104184680@stu.syau.edu.cn (Q.B.); libin@syau.edu.cn (B.L.); 2 College of Bioscience and Biotechnology, Shenyang Agricultural University, Shenyang 110866, China; yanyeruan@syau.edu.cn

**Keywords:** purple corn anthocyanins, microcapsule, maltodextrin, stability, digestion, chewing tablet

## Abstract

Purple corn anthocyanins are important natural colourants with cheap prices and rich bioactivities. However, their stability is limited. Microencapsulation is an effective way to improve anthocyanin stability and the influence of the type of wall material on the stability of encapsulated anthocyanin is very important. In this study, maltodextrin (MD) and its combination with whey protein isolate (WPI) or gum arabic (GA) were utilised as wall materials to obtain encapsulated purple corn anthocyanins (PCAs) (MD–PCA, MD–WPI–PCA, MD–GA–PCA) using spray drying. The effect of the amount of the wall material was determined by encapsulation efficiency, anthocyanin content, and colour. On this basis, the effects of the types of wall materials on the physicochemical characteristics, storage, and digestion stabilities of encapsulated PCA, as well as their stabilities in chewing tablets, were investigated. The highest encapsulation efficiency, suitable colour, and anthocyanin content were obtained with the mass ratios 1:1 PCA to MD, 2:3 PCA to MD–GA, and 1:1 PCA to MD–WPI. Microencapsulation increased PCA storage and digestion stabilities. All three types of PCA microcapsules had low water content and hygroscopicity and good water solubility. MD–PCA had the strongest stability when stored at 25 °C; MD–GA–PCA—when stored at 40 °C, or in the presence of 5000 Lux light illumination; MD–WPI–PCA—when stored in 75% relative humidity or during gastric–intestinal digestion, but its resistance to 40 °C temperature and light illumination was lower than those for the two others. When used in chewing tablets, MD encapsulation was most stable in the presence of Ca^2+^, V_C_, or Fe^2+^ and improved PCA digestion stability. In conclusion, MD is a good choice for PCA encapsulation in regular conditions. MD–GA and MD–WPI can be used when considering high storage temperature (or light illumination) and high humidity (or for high digestion stability), respectively. The results of this study provide a reference for the storage and application of PCA.

## 1. Introduction

Anthocyanins are pigments found in purple, black, and red vegetables, fruits, and grains [1]. Anthocyanins exhibit antioxidant, anticancer, and antidiabetic effects [2]. However, anthocyanins are extremely sensitive to pH, temperature, light, metal ions, and enzymes, which leads to their degradation and low utilisation during storage, processing, and digestion [3]. Encapsulation technologies, such as freeze drying, spray drying, liposome entrapment, and nanoencapsulation, have emerged as effective approaches that entrap anthocyanins inside the wall materials to slow their degradation under various environmental factors during processing and storage and control their release during digestion [3,4]. Among these, spray drying is most commonly used because of its simple operation, high speed, and low cost [5].

The type of wall material is of great importance for the properties of anthocyanin encapsulation, such as encapsulation efficiency, colour, shape, stability, and digestion [6]. Carbohydrates, plant gums, and proteins are the primary wall materials used for encapsulation. Maltodextrin (MD) is the most commonly used wall material for anthocyanin encapsulation because of its good water solubility, high encapsulation efficiency, and low viscosity [7]. It plays numerous roles, including coating and protecting anthocyanins from oxygen and heat [5]. Gum arabic (GA) is another widely used wall material for encapsulation; however, it is usually combined with other materials because of its high cost [5]. Whey protein isolates (WPIs), which have good emulsification, water solubility, and film-forming properties, have recently gained increasing attention as wall materials for bioactive compounds [8]. WPIs have been reported to improve the digestion stability and antioxidant activity of anthocyanins [9]. Norkaew et al. [10] investigated the influences of MD, GA, WPI, and different combination ratios of MD–WPI and MD–GA on the physicochemical properties and stabilities of encapsulated black rice anthocyanins, and found that MD-encapsulated anthocyanins had the highest retention rate (88%), followed by MD–WPI with combination ratios of 3:7 and 1:1, indicating that the effects were dependent on the type and combination ratio of the wall materials and the structure of the anthocyanins.

Purple corn anthocyanins (PCAs) are important natural compounds which can be used as raw materials for food colourants, nutraceuticals, and cosmeceuticals [11]. PCAs have antioxidant, anti-inflammatory, anti-cancer, and anti-mutative properties [12]. However, their stability is limited [13]. Lao and Giusti [14] used MD as a wall material to encapsulate PCA via spray drying and indicated that the yield, anthocyanin retention rate, and water solubility of the encapsulate were influenced by the MD amount, PCA matrix, and inlet temperature. Ren and Giusti [15] found that the addition of whey protein (10 mg/mL) decreased PCA degradation in the presence of Vitamin C (V_C_) and increased the half-time by about two times. Therefore, the choice of encapsulation wall materials for the improvement of PCA stability and their application in industry is urgent. However, the stability of MD-encapsulated PCA under different storage and processing conditions, and the application of a combination of MD and GA (or WPI) for PCA encapsulation, has rarely been reported. 

In this study, MD, GA, and WPI were used to encapsulate PCA. Encapsulated anthocyanins, MD–PCA, MD–GA–PCA, and MD–WPI–PCA were obtained. The effects of the mass ratio of PCA to MD (MD–GA and MD–WPI) on the encapsulation efficiency (EE), anthocyanin content, and colour were studied. The physicochemical properties and morphologies of the encapsulated PCA were determined. The anthocyanin retention rate, colour, free radical scavenging ability, and degradation kinetics were studied to evaluate their storage stability at 25 °C, 40 °C, 5000 Lux light, and 75% relative humidity. In addition, their stability in the digestive tract and in chewing tablets with V_C_, Ca, and Fe was explored. The results will help provide guidance for choosing suitable wall materials to satisfy different environmental requirements, improve the stability of PCA, develop stable PCA-related products, and accelerate PCAs’ utilisation in food and related industries.

## 2. Materials and Methods

### 2.1. Preparation of PCA Extract Solution

Naturally dried purple corn was obtained from the College of bioscience and biotechnology, Shenyang Agricultural University. The seeds were ground into powder and screened using a 40-mesh sieve. The resulting powder was mixed with acidified ethanol (absolute ethanol and 0.2 mol/L of citric acid solution (*v*/*v*, 7:3)) at a ratio of 1:16 for ultrasonic-assisted extraction with an ultrasonic cleaner (KQ-100DE, Kunshan Ultrasonic Instrument Cable Company, Kunshan, China) at 57 °C for 12 min. The anthocyanin extract was then obtained using suction filtration and concentrated with vacuum rotary evaporation (RE-52AA, Shanghai Yarong Biochemical Instrument Factory, Shanghai, China) to remove ethanol (the total soluble solids content was 14.5–16.7%). Vacuum pressure, heating bath temperature, and rotating speed were −1 Mpa, 40 °C, and 80 rpm, respectively. The coolant was flowing tap water (15 °C).

### 2.2. Preparation of PCA Encapsulate

The wall materials used for PCA encapsulation included MD, MD–GA combination (mass ratio 1:1), and MD–WPI combination (mass ratio 3:7). The mass ratios of the combinations were based on the results of Norkaew et al. [10].

The PCA extract solution was mixed with the wall material according to the mass ratio of soluble solids in the PCA extract solution to the wall material. For MD, the ratios were 5:3, 1:1, and 3:5; for MD–GA, the ratios were 1:1, 1:2, and 2:3; and for MD–WPI, the ratios were 1:2, 1:1, and 2:1. 

After mixing, the mixture was homogenised, the concentration of soluble solids was adjusted to 15% with citric acid-sodium citrate buffer solution (0.1 M, pH 3.0), and the mixture was loaded into a spray drier (SD-1500, Shanghai Triowin Technology Company, Shanghai, China). The feed temperature was 60 °C, peristaltic pump speed was 350 r/h, inlet air temperature was 140 °C, and outlet air temperature was 50 °C. After drying, the PCA encapsulates were collected and EE, anthocyanin content, and colour were evaluated to choose the optimal amount of encapsulated wall material for the determination of physicochemical properties and stability.

### 2.3. Encapsulation Efficiency

The method described by Robert et al. [16] was used, with modifications. 

Microcapsules (0.2 g) were dissolved in 10 mL of citric acid-sodium citrate buffer solution (0.1 M, pH 3.0) for determining total PCA content in microcapsules, which included anthocyanin content on microcapsule surface and inside the microcapsule, while 10 mL of absolute ethanol was used to wash the surface of microcapsules to determine PCA on the surface of microcapsules.

Encapsulation efficiency (%) = (1 − anthocyanin content on microcapsule surface/total anthocyanin content in microcapsules) × 100

### 2.4. Colour

Following the method of Bhagya Raj and Dash [17], the colour of the microcapsules was measured using a colourimeter (NH310, Shenzhen Sanenshi Technology Company, Shenzhen, China). The L*, a*, and b* parameters were recorded. L* (brightness) ranged from 0 (black) to 100 (white); +a* (−a*) and +b* (−b*) represent the red (green) and yellow (blue) value, respectively. The total difference (ΔE) was calculated as follows:

$$\Delta E=\sqrt{{\left(L_{0}*-L_{t}*\right)}^{2}+{\left(a_{0}*-a_{t}*\right)}^{2}+{\left(b_{0}*-b_{t}*\right)}^{2}},$$

where L_0_* and L_t_* represent the colour parameters of the microcapsules at the beginning and after t days of storage, respectively, and a_0_* and a_t_*, b_0_*, and b_t_* are expressed in the same manner.

### 2.5. Physical Characteristics of PCA Microcapsules

#### 2.5.1. Moisture Content

According to the method of AOAC [18], microcapsules (1 g) were weighed and dried in an oven (DHG-9070A, Shanghai Jinghong Experimental Equipment, Shanghai, China) at 105 °C (air velocity, 1400 rpm) to constant weight. The moisture content was expressed as the mass percentage of the evaporated water in the microcapsules before drying.

#### 2.5.2. Solubility

Following the description of Shittu and Lawal [19] with slight modifications, microcapsules (0.1 g) were put into distilled water (10 mL, 30 °C) and magnetically agitated (100 rpm) for 30 min, then centrifuged (8000× *g*, 10 min). The resulting supernatant was dried at 105 °C to constant weight. The solubility represents the mass percentage of the dried solid in the microcapsules before drying.

#### 2.5.3. Hygroscopicity

Following the description of Kurek et al. [20], with small modifications, microcapsules (1 g) were stored in a container with saturated sodium chloride solution at 25 °C for one week. Hygroscopicity was the mass of absorbed water per 100 g of microcapsules.

#### 2.5.4. Fluidity 

Fluidity was expressed by the angle of repose (θ) measured as described by Huang et al. [21], with small modifications. Microcapsules (10 g) were poured on the horizontal plane through a funnel to make them naturally stacked, then the height (h) of powder pile and the radius (r) of the co-powder pile were measured, and θ was calculated according to the following formula:

$$\theta \;=\arctan\;\frac{h}{r}$$

### 2.6. Morphology of PCA Microcapsules

After gold spraying, the morphology of the microcapsules was observed using a scanning electron microscope (SEM) HITACHI Regulus8100 (Tokyo, Japan). Gold layer was 10 nm. Multiple magnifications were 1000 and 2000. Accelerating voltage was 3.0 kV. Objective aperture was 50 μm. Working distance was 9.1 mm.

### 2.7. Storage of PCA Microcapsules

PCA microcapsules and freeze-dried unencapsulated PCA (freeze-dried PCA extract solution described in Section 2.1 was used as a control) were placed in conical bottles with stoppers and stored at different temperatures (25 °C, 40 °C) in the dark, 25 °C in the presence of 5000 Lux illumination intensity (Illuminometer, LS-4000uv1000–7000LX, Beijing Star Technology Co., Ltd., Beijing, China), and 25 °C with relative humidity 75% (prepared using a saturated NaCl solution) in the dark for 30 days. Microcapsules (0.1 g) removed after a period of time were used for the determination of colour difference (Section 2.4) or dissolved in 10 mL of citric acid-sodium citrate buffer solution (0.1 M, pH 3.0) with an ultrasonic cleaner (25 °C) for determining total PCA content (pH differential method) [11] and 2,2-diphenyl-1-picrylhydrazyl (DPPH) radical scavenging ability. 

### 2.8. DPPH Free Radical Scavenging Ability

Following the slightly modified method of Chen et al. [22], 0.2 mL sample solution (Section 2.7) was mixed and reacted with 0.2 mL of DPPH ethanol working solution for 30 min at room temperature in the dark, and the absorbance was measured at 517 nm.

### 2.9. Degradation Kinetics of PCA Microcapsules during Storage

First-order kinetics were applied to evaluate anthocyanin degradation under different storage conditions according to Akhavan Mahdavi et al. [23] and Nazareth et al. [24]. The first-order kinetics are expressed by Equation (1), and the half-time (t_1/2_) was calculated using Equation (2):

(1) $$\ln\frac{C_{t}}{C_{0}}=-\mathrm{kt}$$

(2) $$t_{1/2}\;=\frac{\ln\;2}{k}$$

where C_0_ and C_t_ are the anthocyanin content at the start and after t days of storage (mg/g), respectively, t is the storage day (d), and k is kinetic constant (d^−1^).

### 2.10. Preparation and Storage of Chewing Tablets

Formulation of chewing tablets: The weight per tablet was 0.5 g. The amounts for V_C_, ferrous fumarate, and calcium carbonate were 9%, 4.5%, and 37.5% per tablet, respectively, when added alone (not combined) to the tablet. The amounts of microcapsules, magnesium stearate, citric acid, and stevioside were 50%, 1%, 2%, and 0.1%, respectively, while the rest was supplemented by microcrystalline cellulose. 

The preparation of the chewing tablets was based on the description of Wu et al. [25] with some modifications. The materials were weighed, screened through a 100-mesh sieve, and then mixed. A small amount of water was added to the mixture to create a damp mass, which was screened into granules using an 18-mesh sieve. The granules were dried in an oven (DHG-9070A, Shanghai Jinghong Experimental Equipment, Shanghai, China) at 50 °C for two hours, passed through a 20-mesh sieve, and compressed into tablets using a tablet machine (DF-4, Tianjing Port East Technology Development Company, Tianjin, China).

### 2.11. In Vitro Digestion

In vitro digestion was conducted according to the description of Zhang et al. [26], with some modifications. PCA microcapsules and chewing tablets (0.2 g) were dissolved in 50 mL 0.9% NaCl and adjusted to pH 2 with 1 M HCl solution, followed by adding 0.16 g pepsin (activity, 1:3000), then shaken (120 rpm/min) at 37 °C for two hours to stimulate gastric digestion. Thereafter, the pH of the digested solution was adjusted to pH 7.5 with 0.5 M NaHCO_3_, 12 mL of 2 mg/mL trypsin solution and 6 mL of 12 mg/mL bovine bile salt were added, and the mixture was shaken (120 rpm/min) at 37 °C for two hours to stimulate intestinal digestion. During gastric and internal digestion, 1 mL of the digestion solution was collected every 30 min and heated in a boiling water bath for one minute to kill the enzyme, then cooled and centrifuged for 15 min (6000× *g*), and the anthocyanin content in the supernatant was determined.

### 2.12. Statistical Analysis

All experiments were repeated three times, and the data were expressed as mean ± standard deviation. The statistical significance analysis of each factor in the single-factor test was calculated using SPSS Statistics V22.0.

## 3. Results and Discussion

### 3.1. Influence of the Amount of Wall Material on the EE and Colour of PCA

As shown in Table 1, with an increase in the white colour addition to the wall material, the L* and b* values of the microcapsules increased, whereas the a* values decreased; the colour of the microcapsules became increasingly light pink and was not close to that of the unencapsulated PCA. With an increase in the wall material, the anthocyanin content of the microcapsules decreased. Among them, the anthocyanin content in MD–PCA, MD–GA–PCA, and MD–WPI–PCA ranged from 76.01 to 143.39 mg/100 g, 69.61 to 83.25 mg/100 g, and 59.58 to 136.99 mg/100 g, respectively, higher than that (53.27 to 108.46 mg/100 g) of red cabbage anthocyanin microcapsules prepared by MD or MD–GA (combination ratio of 35:15 or 25:25) [27]. With an increase in the wall material, the EE of the PCA increased and then began to decline slightly, which may have been due to the decreased atomisation efficiency of spray drying caused by the high viscosity of the whole system [23]. When the core wall ratio was 1:1 for MD–PCA, 2:3 for MD–GA–PCA, and 1:1 for MD–WPI–PCA, the EE reached a maximum of 94.6%, 93.4%, and 94.4%, respectively, and the colour and anthocyanin content were suitable. Therefore, these three ratios were selected to prepare the microcapsules and used to determine their properties and stabilities. Akhavan Mahdavi et al. [23] found a higher EE of MD–GA (combination ratio of 3:1) on barberry anthocyanin than on MD alone. Norkaew et al. [10] found that the combination of MD with GA or WPI at ratios of 7:3, 1:1, and 3:7 had similar EEs (reached 100%) to that of MD alone on black rice anthocyanin, suggesting that the EE of anthocyanins was influenced by anthocyanin structures, the different amount of the wall materials, and combination ratios of MD to GA or WPI.

### 3.2. Physical Properties of the Three Kinds of Encapsulated PCA

Generally, the food industry requires the moisture content of food powder to not exceed 4% [28]. A low water content can maintain the microcapsules in a glassy state, which can restrict undesirable impacts such as molecular mobility and oxidation [29]. The water contents of the three types of microcapsules were all lower than 4% (Table 2), suggesting high spray-drying efficiency, good storage stability, and overall acceptability of the microcapsule powders [6]. 

Solubility reflects the dissolution rate of microcapsules when they are redissolved in water. The solubility of the three kinds of PCA microcapsules was 79.92% to 81.23%. The order was as follows: MD–PCA > MD–WPI–PCA > MD–GA–PCA (Table 2). Similar results were reported by Sarabandi et al. [30], who found that eggplant peel extract encapsulated by MD had higher solubility than that encapsulated by MD–GA. Pieczykolan and Kurek [7] indicated that capsules with small size and low water content had greater solubility. 

Higher hygroscopicity causes powder agglomeration and affects its fluidity and nutritional value [28]. Ng and Sulaiman [31] believed that under 75% relative humidity, the microencapsulated powder with moisture absorption in the range of 15.1–20.0 g/100 g dry matter has strong hygroscopicity, indicating that the hygroscopicity of the three kinds of microencapsules in this study was weak (6.08~9.56 g/100 g). Moreover, MD–WPI–PCA had the lowest hygroscopicity, while MD–PCA had the highest hygroscopicity, suggesting that MD–WPI–PCA is more conducive to storage under an environment with high humidity. Rajabi et al. [32] indicated that microcapsules produced by wall materials with higher glass transition temperatures had lower hygroscopicity, such as the higher glass transition temperature of GA than that of MD.

The angle of repose, also known as the free-flow angle, was used to analyse the fluidity of finished products; the smaller the angle of repose, the better the fluidity [33]. A product with an angle of repose higher than 45° had poor fluidity [34]. The fluidity ranking of the three types of microcapsules was as follows: MD–PCA > MD–GA–PCA > MD–WPI–PCA. This may be due to the lower moisture content and mutual adhesion forces between the microcapsules [35].

### 3.3. SEM Analysis

**Figure 1:**
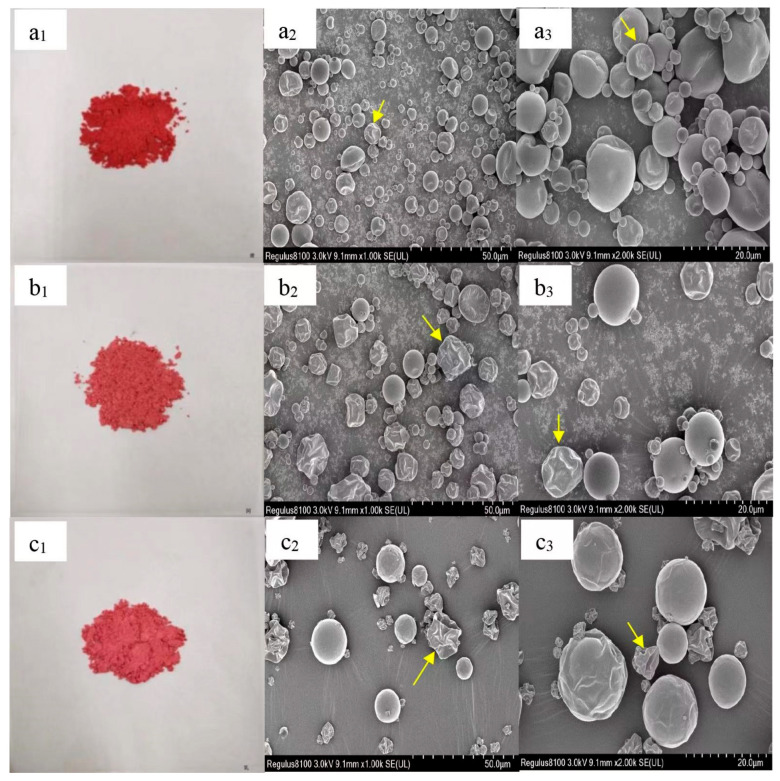
SEM images of MD–PCA, MD–GA–PCA, and MD–WPI–PCA. (**a_1_**–**c_1_**,**a_2_**–**c_2_**,**a_3_**–**c_3_**) are the morphologies observed under the digital camera, the SEM at 1000× multiples, and the SEM at 2000× multiples, respectively. MD–PCA, MD–GA–PCA, and MD–WPI–PCA indicate purple corn anthocyanin encapsulated by maltodextrin, the combination of maltodextrin and gum arabic, and the combination of maltodextrin and whey protein isolate, respectively. Yellow arrows point to microcapsules with concavities and wrinkles.

As shown in Figure 1, the shapes of the three types of microcapsules were spherical. No cracks were observed on the microcapsule surface. The sizes of the MD–PCA microcapsules were 8~80 μm, which was more uniform and relatively smaller than those of MD–GA–PCA (10~80 μm) and MD–WPI (10~100 μm), consistent with the conclusion of Sendri et al. [36] that the size of the microcapsules encapsulated by complex wall materials is larger than that for a single wall material. Most microcapsules had concavities and wrinkles. An increasing number of deeper concavities and wrinkles was found in the microcapsules of black rice anthocyanins encapsulated with MD and their combination with GA or WPI [10]. These were caused by the deformation of the wall materials, resulting from the rapid evaporation of the atomised droplets by the higher temperature of the inlet’s hot air. In addition, lower emulsifying and film-forming abilities can also lead to surface problems in microcapsules [23]. 

### 3.4. Storage Stability Analysis of the Three Kinds of Encapsulated PCA under Different Temperatures

**Figure 2:**
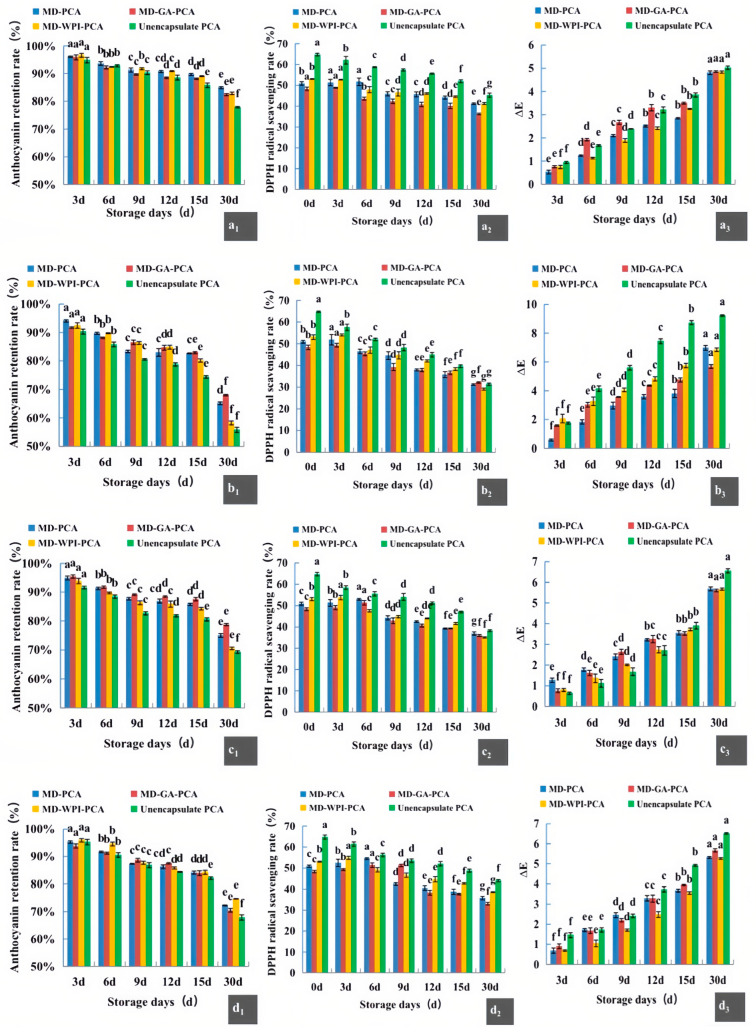
Change in anthocyanin retention rate, antioxidant activity, and ∆E value of unencapsulated PCA, MD–PCA, MD–GA–PCA, and MD–WPI–PCA. (**a_1_**–**a_3_**,**b_1_**–**b_3_**,**c_1_**–**c_3_**,**d_1_**–**d_3_**) are the change at 25 °C, 40 °C in dark, 25 °C in the presence of 5000 Lux illumination intensity, and 25 °C with relative humidity 75% in the dark, for 30 days, respectively. PCA indicates purple corn anthocyanin; MD–PCA, MD–GA–PCA, and MD–WPI–PCA indicate purple corn anthocyanin encapsulated by maltodextrin, the combination of maltodextrin and gum arabic, and the combination of maltodextrin and whey protein isolate, respectively. Different lowercase letters in the same column indicate significant difference (*p* < 0.05).

As shown in Figure 2, with the increase in storage time, the PCA content of all three kinds of microcapsules decreased, and ∆E value became larger due to decreased PCA retention in the microcapsules. However, the DPPH free-radical-scavenging activity initially increased slightly and then decreased. The result was consistent with that of Wu et al. [37], who studied the change in antioxidant activities of blueberry anthocyanin microcapsules encapsulated by WPI–GA during storage, through the determination of ABTS radical scavenging ability and ferric iron reducing antioxidant power (FRAP). They indicated that the small increase may be caused by the phenolic acids formed by the degradation of anthocyanins in the microcapsules in the early stage of storage, which slightly increased the antioxidant capacity, followed by the decomposition of anthocyanins and phenolic acid in the microcapsules in the late stage of storage, when the antioxidant activity began to decline. The degradation of PCA was more obvious at 40 °C since high temperature causes the generation of unstable aglycones resulting from the loss of glycosides in anthocyanins [38].

As shown in Figure 2, compared with unencapsulated PCA (control), the change extent of anthocyanin retention rate, antioxidant activity, and ∆E value of the three kinds of microcapsules were all smaller. Moreover, as shown in Table 3, compared with unencapsulated PCA, the k value of the degradation kinetics of the microcapsules decreased and t_1/2_ increased, suggesting that the wall material protected the PCA from heat and slowed its degradation rate. Among them, MD–PCA showed the best stability at 25 °C (t_1/2_ = 173.29 days), consistent with the results of Kang et al. [39], who compared the stability of chlorophyll encapsulated with MD and its combination with GA. However, MD–GA–PCA had the best stability under 40 °C (t_1/2_ = 69.31 d), suggesting that MD and GA had a good synergistic effect on protecting PCA from degradation under high temperature [6]. Machado et al. [27] indicated that higher thermal stability of the wall material resulted in a stronger stability of its core.

### 3.5. Storage Stability of the Three Kinds of Encapsulated PCA under 5000 Lux Light Intensity

As shown in Figure 2, with the increase in light duration at 25 °C, the PCA retention rate, DPPH free radical scavenging activity, and the ∆E value showed the exact change trends as under different temperatures in the dark. However, light illumination accelerated the degradation of anthocyanin at 25 °C. Table 3 also shows that the degradation rate (k) under light was larger compared with that at 25 °C in dark, indicating that light accelerates the degradation of anthocyanins; thus, the half-life (t_1/2_) decreased. Nie et al. [40] indicated that ultra-violet (UV) light changed anthocyanins from the ground state to the excited state, which then converted into C4 adducts after hydrolysis at the C4 position of the aglycon. 

Among the three kinds of microcapsules, MD–GA–PCA microcapsules had the highest anthocyanin retention and lowest decrease extent of antioxidant activity and increase extent of ∆E value (Figure 2), suggesting that the combination of MD and GA can protect PCA against light more effectively. Akhavan Mahdavi et al. [23] found that barberry anthocyanins encapsulated by MD–GA had higher retention rates than those encapsulated by MD alone. Moreover, Table 3 shows that MD–GA–PCA had the smallest degradation rate (k) and the largest half-life (t_1/2_ = 115.52 d), followed by MD–PCA and MD–WPI–PCA (t_1/2_ = 99.02 and 77.02 d, respectively), whereas the t_1/2_ of unencapsulated PCA was 69.31 d, indicating that MD–GA–PCA had the best protective ability for PCA against light, while the effect of MD–WPI was relatively low, consistent with the results shown in Figure 2. 

### 3.6. Storage Stability of the Three Kinds of Encapsulated PCA under 75% Relative Humidity

As shown in Figure 2, with the increase in duration under 75% relative humidity in the dark, the changing trend of PCA retention rate, DPPH free radical scavenging activity, and the ∆E value showed the same change trends as those under the other three conditions. The degradation rates k of the encapsulated and unencapsulated PCA were higher than those for conditions at 25 °C in the dark without higher humidity, indicating that the humidity can accelerate their degradation. High relative humidity increases the mobility of molecules, thus accelerating the degradation reaction rate [23]. All three types of microcapsules exhibited a lower degradation rate k and a prolonged half-life (t_1/2_), suggesting that the wall materials protected the PCA against humidity damage. 

As shown in Figure 2, among the three kinds of microcapsules, the change extents of PCA retention rate, DPPH free radical scavenging activity, and the ∆E value of MD–WPI–PCA microcapsules were all smaller than those of MD–PCA and MD–GA. It was observed that the microcapsules of MD–PCA and MD–GA–PCA became sticky and caked on the 6th day of storage, while MD–WPI–PCA became slightly sticky by the 15th day but were still in powder form and maintained good fluidity, indicating that the hygroscopicity of MD–WPI–PCA was weaker than that of MD–PCA and MD–GA–PCA. Moreover, Table 3 shows that MD–WPI–PCA had the lowest degradation reaction rate k and longest t_1/2_ compared with the other two wall materials encapsulated in PCA, indicating that MD–WPI had the strongest resistance to humidity and protected PCA against degradation caused by humidity more effectively, which may be due to its lower hygroscopicity [23]. Comparing the degradation rates k and t_1/2_ under 75% humidity with those under other conditions, it was clear that MD–WPI had a stronger tolerance to humidity, whereas MD and MD–GA resisted light better. 

### 3.7. Stabilities of Encapsulated PCA in Chewing Tablets with Ca^2+^, V_C_, and Fe^2+^

**Figure 3:**
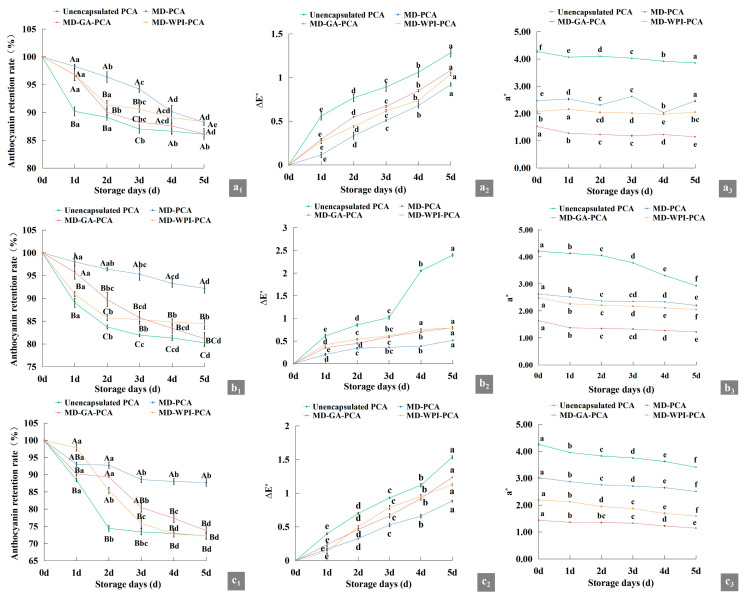
Change in anthocyanin retention rate, ∆E, and a* value of unencapsulated PCA, MD–PCA, MD–GA–PCA, and MD–WPI–PCA in the chewing tablets with Ca^2+^, V_C_, and Fe^2+^. (**a_1_**–**a_3_**,**b_1_**–**b_3_**,**c_1_**–**c_3_**) are the change in the presence of Ca^2+^, V_C_, and Fe^2+^, respectively. Note: PCA indicates purple corn anthocyanin; MD–PCA, MD–GA–PCA, and MD–WPI–PCA indicate purple corn anthocyanin encapsulated by maltodextrin, the combination of maltodextrin and gum arabic, and the combination of maltodextrin and whey protein isolate, respectively. Different lowercase letters in the same line or uppercase letters in the same day indicate significant difference (*p* < 0.05).

As shown in Figure 3, with an increase in storage time, PCA retention rate and the value of a* of the chewing tablets decreased gradually, whereas ΔE increased, suggesting that PCA was unstable in the presence of Ca^2+^, V_C_, and Fe^2+^, similar to the results of Shi and Lv [41], who found that the addition of Ca^2+^ and Fe^3+^ decreased the absorbance of the PCA pigment solution. However, the retention rates of the three kinds of encapsulated PCA were higher than those of unencapsulated PCA; among them, the retention rate of MD–PCA was the highest and that of ΔE was the smallest, suggesting that MD can protect PCA from degradation caused by Ca^2+^, V_C_, and Fe^2+^ more effectively. In particular, the effects of preventing degradation from V_C_ and Fe^2+^ were more obvious (retention rates of PCA 92.20% and 87.75%, respectively) than those of the other two wall materials. In addition, MD–WPI had stronger effects on preventing PCA degradation from Ca^2+^ and V_C_ and a weaker effect on preventing PCA degradation from Fe^2+^ than MD–GA. Ren and Giusti [15] indicated that V_C_ decreased the colour intensity of anthocyanins because of its condensation at the C4 position of anthocyanin and its activation of the oxidative cleavage of the pyrylium ring, and increased addition of WP to the PCA solution protected the destruction caused by V_C_ through the formation of a complex with PCA, which is beneficial for the generation of flavylium cations.

### 3.8. Digestion Stabilities of PCA from Microcapsules and Chewing Tablets

**Figure 4:**
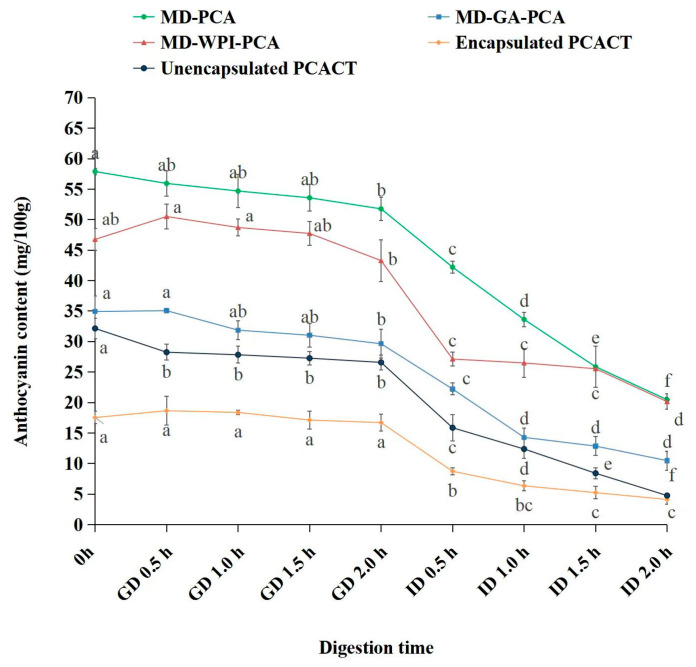
Content change of anthocyanin released from microcapsules (MD–PCA, MD–GA–PCA, MD–WPI–PCA), unencapsulated PCA from the chewing tablets (Unencapsulated PCACT), and MD-encapsulated PCA from the chewing tablets (Encapsulated PCACT) during gastric (GD) and intestinal digestion (ID). Note: MD–PCA, MD–GA–PCA, and MD–WPI–PCA indicate purple corn anthocyanin encapsulated by maltodextrin, the combination of maltodextrin and gum arabic, and the combination of maltodextrin and whey protein isolate, respectively. Different lowercase letters in the same line indicate significant difference (*p* < 0.05).

As shown in Figure 4, at the initial time, the PCA released from the MD-encapsulated microcapsules to the gastric digestion solution reached a maximum, whereas the PCA released from MD–GA and MD–WPI reached a maximum in the digestion solution after gastric digestion (0.5 h), mainly due to the high solubility of MD. This phenomenon was also observed by Norkaew et al. [10], who studied the release of anthocyanins from black rice during digestion. The content of PCA released from the three types of microcapsules during gastric digestion was steady, whereas the extent of change was larger during intestinal digestion, consistent with the results of Righi da Rosa et al. [6], who studied the stimulated digestion of microencapsulated blueberry anthocyanins. Compared with the initial value of anthocyanins in the digestion solution, the retention of MD–PCA, MD–GA–PCA, and MD–WPI–PCA after two hours of gastric digestion was 89.40%, 85.73%, and 92.95%, respectively. After two hours of intestinal digestion, the retention of MD–PCA, MD–GA–PCA, and MD–WPI–PCA decreased to 35.36%, 30.33%, and 43.12%, respectively, suggesting that MD–WPI can protect PCA more effectively during gastric and intestinal digestion, followed by MD and MD–GA. For the anthocyanins from the chewing tablets, the release of encapsulated PCA from the chewing tablets was slower, and its retention was 95.27% and 23.50% after gastric and intestinal digestion, respectively, while the retention of unencapsulated PCA was 82.67% and 14.81%, respectively, suggesting that encapsulation can improve the digestion stability of PCA from the chewing tablets, which was mainly because the wall materials delayed the release of PCA and avoided the degradation caused by the digestion solution. 

## 4. Conclusions

When the mass ratio was 1:1 for PCA to MD, 2:3 for PCA to MD–GA combination (mass ratio of 1:1), and 1:1 for PCA to MD–WPI combination (mass ratio of 3:7), MD–PCA, MD–GA–PCA, and MD–WPI–PCA had the highest EEs (94.6%, 93.4%, and 94.4%, respectively), favourable colour, and anthocyanin content in microcapsules (117.22, 84.08, and 110.81 mg/100 g, respectively). The three types of encapsulated PCA were all spherical. Their sizes ranged from 8 to 100 μm. The moisture content of the three kinds of microcapsules was lower than 4%, their solubility was good (79.92~81.23%), and the hygroscopicity was weak (6.08~9.56 g/100 g). The order of solubility was MD–PCA > MD–WPI–PCA > MD–GA–PCA, whereas the hygroscopicity and fluidity order was MD–PCA > MD–GA–PCA > MD–WPI–PCA. All three types of wall materials improved the PCA stability, whether in storage or during simulated digestion. MD–GA–PCA had the strongest resistance to the storage degradation caused by 40 °C temperature or 5000 Lux light illumination. MD–WPI–PCA showed the strongest resistance to degradation at 75% relative humidity and the highest stability during gastric and intestinal digestion. MD–PCA can maintain the strongest stability at 25 °C or when used in chewing tablets in the presence of Ca^2+^, V_C_, or Fe^2+^, especially the strongest resistance to V_C_ and Fe^2+^. MD–WPI–PCA had stronger resistance to Ca^2+^ and V_C_ but weaker resistance to Fe^2+^ than MD–GA–PCA. The results of this study provide a reference for the application of PCA to storage, digestion, and processing. MD is a good wall material for PCA encapsulation in regular conditions. MD–GA can be used when high temperatures or light illumination are difficult to avoid, and MD–WPI can be considered under very high humidity or for higher digestion stability. In the future, encapsulated PCA will be added to different kinds of food products to investigate their stability and their influence on food qualities.

## Figures and Tables

**Table 1:** Encapsulation efficiency, anthocyanin content, and colour of PCA microcapsules.

PCA Microcapsules	Core Wall Ratio	Encapsulation Efficiency/%	Anthocyanin Content	Colour		
				L*	a*	b*
MD–PCA	3:5	91.64 ± 0.03 ^e^	76.01 ± 0.08 ^g^	65.42 ± 0.04 ^b^	27.47 ± 0.36 ^d^	7.01 ± 0.01 ^b^
	1:1	94.61 ± 0.02 ^a^	117.22 ± 0.11 ^c^	60.87 ± 0.02 ^e^	30.53 ± 0.13 ^a^	4.71 ± 0.03 ^d^
	5:3	81.50 ± 0.05 ^h^	143.39 ± 0.05 ^a^	60.21 ± 0.12 ^f^	30.15 ± 0.19 ^b^	4.66 ± 0.04 ^d^
MD–GA–PCA	1:2	90.67 ± 0.07 ^f^	69.61 ± 0.02 ^h^	64.07 ± 0.08 ^c^	24.48 ± 0.23 ^g^	8.09 ± 0.21 ^a^
	2:3	93.40 ± 0.01 ^c^	84.08 ± 0.06 ^e^	54.52 ± 0.09 ^h^	25.33 ± 0.24 ^f^	5.33 ± 0.06 ^c^
	1:1	64.34 ± 0.02 ^i^	83.25 ± 0.03 ^f^	53.95 ± 0.23 ^i^	28.09 ± 0.08 ^c^	4.59 ± 0.08 ^d^
MD–WPI–PCA	1:2	92.01 ± 0.05 ^d^	59.58 ± 0.05 ^i^	69.84 ± 0.13 ^a^	20.38 ± 0.16 ^i^	5.18 ± 0.09 ^c^
	1:1	94.41 ± 0.01 ^b^	110.81 ± 0.01 ^d^	61.31 ± 0.02 ^d^	23.31 ± 0.03 ^h^	2.03 ± 0.02 ^e^
	2:1	81.90 ± 0.02 ^g^	136.99 ± 0.04 ^b^	58.57 ± 0.23 ^g^	25.74 ± 0.02 ^e^	1.89 ± 0.05 ^e^

Note: PCA indicates purple corn anthocyanin; MD–PCA, MD–GA–PCA, and MD–WPI–PCA indicate purple corn anthocyanin encapsulated by maltodextrin, the combination of maltodextrin and gum arabic, and the combination of maltodextrin and whey protein isolate, respectively. Different lowercase letters in the same column indicate significant difference (*p* < 0.05).

**Table 2:** Physical properties of PCA microcapsules.

PCA Microcapsules	Water Content/%	Solubility/%	Hygroscopicity/ (g/100 g)	Angle of Repose/°
MD–PCA	3.34 ± 0.22 ^c^	81.23 ± 0.15 ^a^	9.56 ± 0.15 ^a^	35.62 ± 0.14 ^c^
MD–GA–PCA	3.56 ± 0.18 ^b^	76.16 ± 0.09 ^c^	7.12 ± 0.03 ^b^	38.85 ± 0.21 ^b^
MD–WPI–PCA	3.82 ± 0.09 ^a^	79.92 ± 0.16 ^b^	6.08 ± 0.24 ^c^	40.09 ± 0.18 ^a^

Note: PCA indicates purple corn anthocyanin; MD–PCA, MD–GA–PCA, and MD–WPI–PCA indicate purple corn anthocyanin encapsulated by maltodextrin, the combination of maltodextrin and gum arabic, and the combination of maltodextrin and whey protein isolate, respectively. Different lowercase letters in the same column indicate significant difference (*p* < 0.05).

**Table 3:** Degradation kinetics constant (k) and half-time (t_1/2_) of PCA microcapsules under different storage conditions.

Storage Condition	PCA Microcapsules	k/d^−1^	R^2^	t_1/2_/d
25 °C, in dark	MD–PCA	0.004	0.955	173.29
	MD–GA–PCA	0.005	0.928	138.63
	MD–WPI–PCA	0.005	0.966	138.63
	Unencapsulated PCA	0.006	0.994	115.52
40 °C, in dark	MD–PCA	0.012	0.979	57.76
	MD–GA–PCA	0.010	0.992	69.31
	MD–WPI–PCA	0.013	0.969	53.32
	Unencapsulated PCA	0.014	0.983	49.51
25 °C, 5000 Lux illumination	MD–PCA	0.007	0.983	99.01
	MD–GA–PCA	0.006	0.940	115.52
	MD–WPI–PCA	0.009	0.981	77.02
	Unencapsulated PCA	0.010	0.973	69.31
25 °C, relative humidity 75%	MD–PCA	0.008	0.987	86.64
	MD–GA–PCA	0.009	0.994	77.02
	MD–WPI–PCA	0.007	0.959	99.02
	Unencapsulated PCA	0.010	0.996	69.31

Note: PCA indicates purple corn anthocyanin; MD–PCA, MD–GA–PCA, and MD–WPI–PCA indicate purple corn anthocyanin encapsulated by maltodextrin, the combination of maltodextrin and gum arabic, and the combination of maltodextrin and whey protein isolate, respectively.

## Data Availability

Data is contained within the article.
